# Supplementary material for: Influence of Inherited Seizure Susceptibility on Intermittent Voluntary Alcohol Consumption and Alcohol Withdrawal Seizures in Genetically Epilepsy-Prone Rats (GEPR-3s)
Source: Brain Sci. 2024 Feb 19;14(2):188. doi: 10.3390/brainsci14020188 (PMC10886844; doi:10.3390/brainsci14020188)
Supplement: Supplementary file 1 [file brainsci-14-00188-s001.zip › brainsci-2873060-supplementary.pdf]

Table S1. Body weights of rats during voluntary alcohol consumption.

	1º week			2º week			3 º week			4 º week			5 º week			6 º week			7 º week		
	Mon	Wed	Fri	Mon	Wed	Fri	Mon	Wed	Fri	Mon	Wed	Fri	Mon	Wed	Fri	Mon	Wed	Fri	Mon	Wed	Fri
GEPR-3s male																					
GEPR 177M	300g	305g	307g	317g	320g	324g	338g	340g	338g	350g	347g	348g	362g	364g	364g	377g	375g	375g	390g	391g	396g
GEPR 178M	310g	313g	316g	326g	326g	323g	329g	333g	335g	343g	346g	347g	359g	357g	356g	365g	361g	368g	386g	390g	395g
GEPR 179M	289g	296g	300g	315g	319g	320g	336g	334g	338g	343g	343g	341g	356g	360g	360g	371g	373g	374g	393g	398g	400g
GEPR 180M	296g	299g	303g	316g	321g	324g	339g	337g	337g	351g	350g	350g	365g	362g	366g	372g	372g	377g	398g	400g	405g
GEPR 181M	301g	305g	309g	315g	318g	327g	331g	336g	340g	349g	352g	347g	361g	360g	362g	373g	370g	376g	402g	404g	406g
GEPR 182M	305g	308g	311g	320g	325g	321g	335g	334g	339g	345g	348g	350g	368g	370g	359g	370g	374g	374g	398g	399g	398g
GEPR 189M	310g	316g	320g	328g	325g	328g	337g	340g	342g	350g	351g	354g	366g	365g	363g	369g	371g	378g	401g	400g	400g
GEPR 190M	297g	301g	305g	312g	316g	322g	330g	333g	336g	352g	350g	353g	363g	365g	368g	373g	371g	375g	396g	397g	399g
GEPR 192M	295g	299g	306g	314g	314g	320g	336g	339g	338g	338g	342g	346g	361g	360g	362g	370g	369g	369g	399g	401g	400g
GEPR 193M	300g	305g	308g	315g	317g	319g	337g	337g	335g	340g	339g	341g	357g	353g	359g	365g	369g	372g	397g	396g	400g
GEPR 194M	298g	304g	310g	318g	323g	324g	332g	335g	338g	344g	348g	347g	364g	364g	363g	373g	372g	375g	404g	407g	405g
GEPR 200M	313g	316g	319g	323g	326g	323g	335g	335g	341g	347g	351g	350g	366g	359g	361g	374g	374g	378g	396g	399g	403g
GEPR-3s female																					
GEPR 183F	230g	230g	232g	242g	244g	244g	257g	257g	259g	272g	276g	274g	280g	279g	283g	291g	290g	290g	301g	299g	305g
GEPR 184F	240g	238g	240g	250g	252g	250g	266g	264g	266g	278g	280g	277g	282g	278g	280g	289g	290g	292g	309g	309g	310g
GEPR 185F	235g	236g	238g	248g	250g	251g	260g	257g	259g	275g	276g	277g	285g	287g	285g	293g	291g	293g	303g	305g	305g
GEPR 186F	232g	235g	237g	247g	249g	250g	263g	260g	262g	277g	279g	280g	290g	293g	290g	297g	298g	300g	310g	310g	309g
GEPR 187F	241g	239g	238g	250g	250g	251g	264g	262g	264g	276g	278g	277g	283g	281g	287g	297g	297g	299g	300g	301g	299g
GEPR 188F	240g	240g	239g	251g	250g	253g	262g	261g	265g	273g	271g	273g	285g	282g	283g	300g	299g	301g	312g	312g	309g
GEPR 189F	229g	231g	234g	247g	244g	245g	259g	260g	259g	269g	272g	274g	279g	280g	284g	289g	290g	293g	299g	301g	304g
GEPR 195F	227g	230g	233g	251g	249g	250g	263g	262g	263g	271g	269g	271g	283g	285g	281g	299g	300g	298g	309g	307g	307g
GEPR 196F	232g	234g	236g	253g	250g	253g	264g	266g	268g	276g	279g	282g	286g	282g	286g	297g	297g	296g	310g	309g	310g
GEPR 197F	235g	236g	235g	242g	246g	254g	258g	260g	257g	270g	274g	276g	290g	289g	290g	301g	300g	301g	313g	313g	311g
GEPR 198F	231g	240g	241g	253g	252g	255g	265g	264g	261g	277g	274g	277g	280g	297g	295g	296g	299g	298g	305g	304g	307g
GEPR 199F	228g	231g	233g	249g	251g	250g	262g	260g	260g	275g	278g	277g	283g	282g	280g	300g	303g	297g	309g	307g	309g
SD male																					
SD 268M	298g	397g	305g	321g	322g	322g	342g	342g	341g	352g	351g	351g	361g	361g	363g	378g	377g	377g	395g	391g	400g
SD 269M	310g	313g	310g	322g	321g	320g	338g	338g	340g	351g	350g	352g	362g	360g	359g	369g	368g	370g	390g	393g	405g
SD 282M	312g	311g	316g	324g	324g	323g	339g	340g	338g	349g	350g	350g	361g	359g	357g	376g	377g	376g	395g	398g	400g
SD 283M	298g	297g	303g	315g	319g	319g	331g	333g	331g	349g	348g	347g	359g	358g	359g	375g	376g	377g	400g	400g	405g
SD 284M	302g	305g	304g	319g	315g	318g	334g	335g	333g	352g	354g	356g	364g	363g	362g	370g	372g	375g	398g	402g	406g
SD 285M	292g	300g	307g	319g	315g	316g	336g	338g	335g	349g	347g	349g	361g	363g	361g	373g	375g	374g	408g	403g	406g
SD 286M	298g	316g	312g	321g	323g	320g	335g	337g	337g	353g	356g	354g	367g	369g	370g	379g	380g	379g	401g	400g	399g
SD 287M	387g	301g	306g	323g	319g	321g	333g	336g	336g	352g	350g	353g	365g	364g	363g	374g	377g	376g	398g	397g	399g
SD 288M	300g	299g	304g	325g	321g	323g	338g	340g	337g	349g	348g	350g	362g	364g	366g	378g	380g	381g	400g	401g	400g

Table S1. Body weights of rats during voluntary alcohol consumption.

SD 289M	396g	303g	308g	323g	325g	325g	335g	340g	337g	347g	348g	349g	369g	370g	369g	376g	379g	380g	405g	402g	407g
SD 290M	298g	304g	310g	324g	320g	324g	336g	339g	339g	349g	350g	349g	367g	369g	369g	378g	382g	381g	397g	400g	398g
SD 291M	306g	308g	312g	320g	323g	323g	340g	342g	341g	350g	350g	348g	360g	363g	366g	368g	370g	373g	399g	397g	401g
SD female																					
SD 271F	236g	234g	234g	245g	242g	243g	258g	260g	260g	272g	272g	273g	283g	282g	281g	295g	297g	298g	310g	306g	315g
SD 272F	229g	230g	231g	243g	243g	246g	260g	261g	263g	270g	273g	272g	280g	282g	283g	289g	292g	296g	302g	301g	301g
SD 276F	230g	229g	230g	242g	243g	244g	254g	256g	260g	272g	271g	272g	283g	282g	283g	289g	289g	290g	299g	301g	304g
SD 277F	234g	232g	231g	245g	244g	246g	260g	259g	258g	273g	270g	271g	284g	283g	285g	290g	291g	291g	298g	302g	303g
SD 278F	240g	240g	238g	241g	243g	247g	258g	259g	260g	269g	270g	271g	285g	284g	285g	291g	293g	294g	299g	300g	302g
SD 279F	228g	230g	234g	239g	242g	245g	256g	260g	258g	270g	271g	273g	284g	285g	284g	292g	291g	293g	302g	301g	301g
SD 292F	226g	229g	233g	241g	244g	246g	257g	260g	259g	271g	272g	272g	283g	284g	283g	290g	293g	293g	300g	300g	299g
SD 293F	233g	235g	237g	245g	247g	246g	259g	261g	262g	273g	272g	274g	282g	283g	285g	293g	295g	294g	298g	399g	298g
SD 294F	240g	245g	242g	248g	247g	248g	258g	263g	262g	271g	270g	273g	284g	286g	286g	295g	294g	293g	300g	300g	301g
SD 295F	234g	236g	239g	246g	246g	247g	260g	259g	260g	274g	275g	274g	286g	286g	288g	296g	295g	296g	303g	305g	308g
SD 296F	229g	228g	233g	243g	244g	246g	254g	257g	256g	270g	273g	274g	282g	284g	287g	294g	293g	295g	305g	307g	311g
SD 297F	228g	230g	234g	241g	243g	245g	256g	258g	257g	269g	271g	275g	286g	288g	290g	296g	295g	296g	302g	304g	309g
